# Recommendations for recognizing, risk stratifying, treating, and managing children and adolescents with hypoglycemia

**DOI:** 10.3389/fendo.2024.1387537

**Published:** 2024-06-04

**Authors:** Stefano Zucchini, Stefano Tumini, Andrea Enzo Scaramuzza, Riccardo Bonfanti, Maurizio Delvecchio, Roberto Franceschi, Dario Iafusco, Lorenzo Lenzi, Enza Mozzillo, Stefano Passanisi, Claudia Piona, Ivana Rabbone, Novella Rapini, Andrea Rigamonti, Carlo Ripoli, Giuseppina Salzano, Silvia Savastio, Riccardo Schiaffini, Angela Zanfardino, Valentino Cherubini

**Affiliations:** ^1^ Study Group of Diabetology of the Italian Society for Pediatric Endocrinology and Diabetes (I.S.P.E.D.,) University Hospital of Ferrara, Ferrara, Italy; ^2^ Department of Maternal and Child Health, UOSD Regional Center of Pediatric Diabetology, Annunziata Hospital, Chieti, Italy; ^3^ Division of Pediatrics, Pediatric Diabetes, Endocrinology and Nutrition, Azienda Socio Sanitaria Territoriale (ASST) Cremona, Cremona, Italy; ^4^ UO Pediatric Diabetes Research Institute, Ospedale San Raffaele, Milan, Italy; ^5^ Department of Biotechnological and Applied Clinical Sciences, University of L’Aquila, L’Aquila, Italy; ^6^ Department of Pediatrics, S. Chiara Hospital of Trento, APSS, Trento, Italy; ^7^ Department of Woman, Child and General and Specialistic Surgery, Regional Center of Pediatric Diabetes, University of Campania ‘L. Vanvitelli’, Naples, Italy; ^8^ Diabetology Unit, Pediatric Department, Anna Meyer Children’s Hospital, Florence, Italy; ^9^ Section of Pediatrics, Regional Center of Pediatric Diabetes, University Federico II, Naples, Italy; ^10^ Department of Human Pathology of Adulthood and Childhood G. Barresi, University of Messina, Messina, Italy; ^11^ Pediatric Diabetes and Metabolic Disorders Unit, Regional Center for Pediatric Diabetes, Department of Surgery, Dentistry, Pediatrics, and Gynecology, University of Verona, Verona, Italy; ^12^ Division of Pediatrics, Department of Health Sciences, University of Piemonte Orientale, Novara, Italy; ^13^ Diabetes Unit, Bambino Gesú Childrens’ Hospital, Rome, Italy; ^14^ Pediatric Diabetology Unit, Department of Pediatrics, ASL 8 Cagliari, Cagliari, Italy; ^15^ Department of Women’s and Children’s Health, Azienda Ospedaliero-Universitaria, Ospedali Riuniti di Ancona, ‘Salesi Hospital’, Ancona, Italy

**Keywords:** adolescents, automated insulin delivery, children, hypoglycemia, glucagon, oral glucose, type 1 diabetes

## Abstract

There has been continuous progress in diabetes management over the last few decades, not least due to the widespread dissemination of continuous glucose monitoring (CGM) and automated insulin delivery systems. These technological advances have radically changed the daily lives of people living with diabetes, improving the quality of life of both children and their families. Despite this, hypoglycemia remains the primary side-effect of insulin therapy. Based on a systematic review of the available scientific evidence, this paper aims to provide evidence-based recommendations for recognizing, risk stratifying, treating, and managing patients with hypoglycemia. The objective of these recommendations is to unify the behavior of pediatric diabetologists with respect to the timely recognition and prevention of hypoglycemic episodes and the correct treatment of hypoglycemia, especially in patients using CGM or advanced hybrid closed-loop systems. All authors have long experience in the specialty and are members of the Italian Society of Pediatric Endocrinology and Diabetology. The goal of treating hypoglycemia is to raise blood glucose above 70 mg/dL (3.9 mmol/L) and to prevent further decreases. Oral glucose at a dose of 0.3 g/kg (0.1 g/kg for children using “smart pumps” or hybrid closed loop systems in automated mode) is the preferred treatment for the conscious individual with blood glucose <70 mg/dL (3.9 mmol/L), although any form of carbohydrate (e.g., sucrose, which consists of glucose and fructose, or honey, sugary soft drinks, or fruit juice) containing glucose may be used. Using automatic insulin delivery systems, the oral glucose dose can be decreased to 0.1 g/kg. Practical flow charts are included to aid clinical decision-making. Although representing the official position of the Italian Society of Pediatric Endocrinology and Diabetology (ISPED), these guidelines are applicable to the global audience and are especially pertinent in the era of CGM and other advanced technologies.

## 1 Introduction

There has been continuous progress in diabetes management over the last few decades. The widespread dissemination and adoption of continuous glucose monitoring (CGM) and automated insulin delivery (AID) systems have completely changed the daily lives of patients with diabetes, improving the quality of life of both children and their families. Despite this, hypoglycemia remains the primary side-effect of insulin therapy. The American Diabetes Association defines hypoglycemia as “all episodes of an abnormally low plasma glucose concentration that expose the individual to potential harm”, highlighting both the physical and psychological/psychosocial dangers. In addition to the adverse effects (i.e., need to interrupt daily activities, unpleasant symptoms, social embarrassment, loss of consciousness, seizures), hypoglycemia may lead to the development of fear of hypoglycemia (FoH). Hypoglycemia and FoH represent the main obstacles to achieving optimal metabolic control. Therefore, the primary objectives of hypoglycemia management for the diabetes care team are timely recognition of hypoglycemic episodes, their prevention, and administering the correct therapy.

The objective of this work is to unify the behavior of all Italian pediatric diabetologists with respect to the timely recognition and prevention of hypoglycemic episodes and the correct treatment of hypoglycemia, also focusing on the psychophysical wellbeing of patients and their families. To achieve this, the Study Group on Diabetes of the Italian Society of Pediatric Endocrinology and Diabetology (ISPED) decided to draw up this set of recommendations on “Hypoglycemia in Children and Adolescents with Diabetes”. The evidence presented within these recommendations is based on a systematic review of the available scientific evidence and a Delphi consensus methodology that involved all participants listed as authors and in the Appendix. All relevant papers published in the last 5 years (January 1^st^, 2019 to December 31^st^, 2023) were carefully evaluated by all authors and were used to inform each section of these recommendations.

In 2022, the International Society for Pediatric and Adolescent Diabetes ISPAD released their Clinical Practice Consensus Guidelines on the assessment and management of hypoglycemia in children and adolescents with diabetes (1). Although these recommendations align with ISPAD, we also provide additional information on rapidly evolving areas of clinical interest including fear of hypoglycemia (FoH), nasal glucagon use, and educational support to fully address the needs of the local (Italian) context.

The ISPED Advisory Council approved this document, and it therefore represents its official position.

## 2 Defining hypoglycemia and its incidence

### 2.1 Definition

The American Diabetes Association defines hypoglycemia in diabetes non-numerically as “all episodes of an abnormally low plasma glucose concentration that expose the individual to potential harm” (2). Nevertheless, it is essential to identify a level of hypoglycemia that should be avoided due to its immediate and long-term impact on the individual (1). Numerical definitions (**Table 1**) are based on glucose values detected by self-monitoring blood glucose (SMBG), CGM, or laboratory measurement of plasma (2). Hypoglycemia is *symptomatic* when the child, adolescent, or parent notices the presence of one or more symptoms and verifies that the blood glucose is <70 mg/dL (3.9 mmol/L).

**Table 1:** Definition of hypoglycemia and clinical targets for CGM data.

Definition	Clinical hypoglycemia alert	Clinically important hypoglycemia	Severe hypoglycemia
**Threshold**	<3.9 mmol/L or <70 mg/dL	<3.0 mmol/L or <54 mg/dL	No specific threshold but linked to symptoms
**CGM target for hypoglycemia**	<4% or <1 hour/day	<1% or <15 min/day	

A hypoglycemic episode can be defined as:

1) <70 mg/dL (3.9 mmol/L): clinical hypoglycemia alert, used as the threshold value for identifying and treating hypoglycemia.
2) <54 mg/dL (3.0 mmol/L): clinically important or serious hypoglycemia. Neurogenic symptoms and cognitive dysfunction occur below this level, together with an increased risk of severe hypoglycemia (3, 4).
3) Severe hypoglycemia: event characterized by altered mental and/or physical status (including coma and seizures) that requires assistance for resolution.

Note that as young children require assistance to correct even mild hypoglycemia, the event requires the caregiver and physician to evaluate whether the child has hypoglycemia-induced cognitive dysfunction.

### 2.2 Incidence

Mild hypoglycemia is common and asymptomatic events are likely to be underreported, making the exact incidence of hypoglycemia difficult to establish. However, symptomatic hypoglycemia is estimated to occur on average twice a week in >80% of people with diabetes, with countless episodes in a lifetime (5). There has been a significant reduction in the incidence rates of hypoglycemia in international registries over the last two decades (6–8).

## 3 Signs and symptoms

The signs and symptoms of hypoglycemia in people living with diabetes (PWD) are caused by adrenergic activation when whole blood glucose falls to 65-70 mg/dL (3.6-3.9 mmol/L) and neuroglycopenia due to glucose deprivation in the brain (9) (**Table 2**).

**Table 2:** Hypoglycemia signs and symptoms (3) (adapted from Abraham MB, ref. 10).

**Autonomic**	Shakiness, sweatiness, trembling palpitations, pallor
**Neuroglycopenic**	Poor concentration Blurred or double vision, disturbed color vision Difficulty hearing Slurred speech Poor judgment and confusion, problems with short-term memory Dizziness and unsteady gait Loss of consciousness, seizure, death
**Behavioral**	Irritability, erratic behavior, agitation, nightmares, inconsolable crying
**Non-specific**	Hunger, headache, nausea, tiredness

The plasma glucose threshold for activation of counter-regulatory hormone secretion is thought to be higher than for initiation of autonomic warning symptoms (~70 mg/dL vs. ~60 mg/dL, respectively) (4). However, a recent systematic review (11) challenged this assumption, with release of counter-regulatory hormones in young adults with type 1 diabetes (T1D) occurring at a median plasma glucose level of 50-61 mg/dL and generation of both autonomic and neuroglycopenic hypoglycemic symptoms starting at a similar glucose level of around 54 mg/dL. These values are lower than those of non-diabetic subjects.

Within the first year of T1D, glucagon responses to hypoglycemia are blunted but epinephrine responses are not; defective and absent glucagon responses to hypoglycemia have been observed in PWD with significant residual endogenous β-cell function (12, 13). In children with T1D, the coalescence of autonomic and neuroglycopenic symptoms may indicate that both responses are generated at similar glycemic thresholds (14). Neuroglycopenic symptoms were reported more commonly in PWD who reported partial awareness of hypoglycemia than those who reported normal hypoglycemia awareness; by contrast, autonomic symptoms were reported less frequently by PWD who had hypoglycemia unawareness (15). Young children typically do not have hypoglycemia awareness or do not have the vocabulary to describe how they are feeling, so it is important to be vigilant for behavioral changes or signs (such as pallor) associated with hypoglycemia (14). The glycemic threshold for hypoglycemia symptoms may occur at a different glucose level in children for different reasons (**Table 3**).

**Table 3:** Variables that influence threshold for onset of symptoms.

**Age**	Symptoms of hypoglycemia and physiological counter-regulatory hormone release occur at a higher glucose level in children than in adults (16).
**Duration of diabetes**	The symptoms of hypoglycemia lessen with increasing duration of diabetes. There is a progressive loss of glucagon response to insulin-induced hypoglycemia over the 12 months after diabetes onset and it is lost in most people with T1D by 5 years (17, 18). Thus, half of adult patients with long-term diabetes have experienced unawareness of hypoglycemia, leading to severe episodes (19), while no data are available for children and adolescents.
**Chronic hyperglycemia**	Chronic hyperglycemia and poor glycemic control can result in an adaptive shift of the threshold of onset for hypoglycemic symptoms to a higher glucose level (20) than in persons without diabetes (21), up to normoglycemic levels (16).
**Recent hypoglycemia**	Glycemic thresholds for symptoms of hypoglycemia shift to lower plasma glucose concentrations after recent antecedent hypoglycemia (22). Hypoglycemia begets hypoglycemia, and recurrent episodes of mild hypoglycemia contribute to the development of defective counter-regulatory hormone responses to subsequent reductions in blood glucose levels (23).

### 3.1 Severe hypoglycemia

In pediatric PWD, severe hypoglycemia is characterized by convulsions, coma, or other neurological symptoms of neuroglycopenia, and it requires therapy with glucagon or IV glucose (17). Risk factors for severe hypoglycemia are age, diabetes duration, glycemic control, type of treatment, unawareness, nighttime episodes, exercise, and previous episodes (10). Other possible risk factors include risky behaviors (alcohol, recreational substances, lack of preparation for sports, infrequent blood glucose monitoring, etc.), as described further below. While younger children are thought to be at higher risk of severe hypoglycemia (24), some studies have not confirmed this association (25–27). Severe hypoglycemia is probably more frequent in adolescents because of the longer duration of disease and higher insulin requirements. Low HbA1c is no longer considered a risk factor for severe hypoglycemia in young PWD since the advent of AID, but a low HbA1c value must always be carefully considered in the clinical context; on the contrary, severe hypoglycemia is followed by a progressive and lasting increase in HbA1c in children and adolescents with T1D (26). People with T1D treated with five or more daily insulin injections were shown to be at reduced risk of severe hypoglycemia compared with subjects on fewer daily injections (24). People with T1D on insulin pumps are at reduced risk of severe hypoglycemia (16), and no episodes of severe hypoglycemia were observed in most hybrid closed-loop (HCL) or advanced hybrid closed-loop (AHCL) trials (18, 19, 28).

## 4 Risk factors

There are several non-modifiable risk factors for hypoglycemia, including younger age, long duration of diabetes, and comorbidities. Several risk factors are, however, modifiable, for example the type of insulin treatment and insulin doses, physical activity, dietary habits, drug use or substance abuse, and others.

### 4.1 Alcohol and hypoglycemia

Alcohol use is common in adolescents with T1D. Since alcohol intake is more frequent during evening hours and plasma glucose mainly decreases 8-12 hours after ethanol administration (20), the risk of hypoglycemia is higher during subsequent sleep hours. Binge drinking represents one end of the spectrum of alcohol consumption, and it is more frequent in males (21). In the DPV registry of youths and young adults with T1D, alcohol use was associated with worse glycemic control, more severe hypoglycemia, and increased rates of diabetic ketoacidosis (DKA) (22).

Even though the combination of alcohol intake and fasting is assumed to induce hypoglycemia, there are few real-time studies on the topic. Garcia et al. (23) reported that moderate alcohol consumption (0.7 g of alcohol per kg of body weight, given as beer) with a mixed meal does not seem to increase the risk of postprandial hypoglycemia over at least six hours post-ingestion. A systematic review applying GRADE criteria by Tetzschner et al. (29) examined studies of alcohol-induced hypoglycemia in PWD and found that most recommendations on hypoglycemia prevention strategies were based on best clinical practice rather than on objective evidence. Overall, the first advice for subjects with T1D is to take precautions when consuming alcohol: the best prevention for alcohol-induced hypoglycemia is, in fact, an awareness of its hypoglycemic effects, especially when it is drunk without simultaneous ingestion of carbohydrates.

### 4.2 Exercise

Exercise can increase the risk of hypoglycemia via several mechanisms: increased glucose consumption, depletion of glycogen stores, increased insulin sensitivity, and exercise-induced counterregulatory hormone deficits (30). The increased risk of hypoglycemia and FoH are a barrier to exercise for PWD (31). The risk of hypoglycemia is greater with aerobic or endurance exercises than with anaerobic or high-intensity exercises (32). The intensity, duration, and type of physical activity, timing to and site of insulin infusion/injection, carbohydrate intake, glucose profile pre-exercise, type of insulin, insulin treatment (MDI/CSII/AID), hydration status, level of training, and age influence the personal risk of hypoglycemia during exercise (33, 34). The risk of hypoglycemia increases with moderate-intensity exercise, immediately after activity, and 7 to 12 hours after exercise (35).

The risk of hypoglycemia is further increased by the reduced counter-regulatory response induced by exercise itself and during sleep (36). Optimizing the glucose profile is fundamental: the appropriate reduction in insulin (basal and bolus insulins) before and after exercise, adequate intake of carbohydrate (before and after exercise), and monitoring of glucose profiles, recognizing that all glucose sensors are less accurate during exercise and in the hypoglycemic range (35).

Another strategy to minimize exposure to hypoglycemia after exercise is to plan exercise sessions at high intensity: counter-regulatory hormones can, in fact, increase endogenous glucose production and maintain glycemia at a higher range than sessions of moderate-to-intense exercise alone (37).

### 4.3 Nocturnal hypoglycemia

Nocturnal hypoglycemia has always been scary for PWD and their parents, significantly affecting their sleep quality and, consequently, quality of life (38). New long-acting insulins have significantly reduced the risk of nocturnal hypoglycemia, especially when compared to NPH insulins. More specifically, degludec and U-300 glargine best prevent nocturnal hypoglycemia in children (39). Furthermore, advances in CGM technology and alarm systems have significantly reduced the risk of hypoglycemia at night (40). The use of HCL with low-glucose suspend (LGS) and the possibility of establishing a specific sleep-hours algorithm have significantly diminished the risk of hypoglycemia (41).

### 4.4 Others

In case of unexplained and repeated hypoglycemic episodes (42), celiac disease (43), Addison’s disease (44), hypothyroidism (45), and factitious hypoglycemia (including Munchausen-by-proxy) should be considered. However, the evidence that subclinical hypothyroidism is a cause of repeated hypoglycemia is weak.

## 5 Treatment of severe hypoglycemia

The primary goal of treatment is to raise blood glucose above 70 mg/dl and to prevent further decreases in blood glucose levels (42). After a severe hypoglycemia episode, it is essential to discuss the reasons why the episode occurred with the subject and caregivers. In addition, close follow-up and regular glucose monitoring are necessary in the days and weeks after the episode.

### 5.1 In-hospital treatment

In a hospital setting, intravenous glucose must be immediately administered to maximally limit exposure to hypoglycemia. The recommended dose of dextrose (glucose) is 0.2 g/kg; this dose can reverse hypoglycemia without the risk of unintentional osmotic diuresis (2). It is essential to pay attention to the concentration of glucose solution and the infusion rate. Highly concentrated glucose solutions (dextrose 50%) or infusion rates >5 mg/kg/min should be avoided due to the risk of excessive rate of osmotic change and, consequently, the risk of hyperosmolar cerebral injury (46). Moreover, highly concentrated glucose solutions can cause peripheral vein sclerosis, so administering glucose solutions at concentrations greater than 25% dextrose is not recommended. 10% dextrose has been shown to be effective and safe for treating hypoglycemia in a randomized controlled trial (47). The recommended dose of dextrose (0.2 g/kg) equals 2 mL/kg 10% dextrose solution. The maximum dose is 0.5 g/kg of body weight, corresponding to 5 mL/kg. In cases of recurrent hypoglycemia with the inability to take an adequate amount of carbohydrates orally, it is possible to prolong the intravenous infusion of 10% dextrose with a glucose infusion rate of 2-5 mg/kg/min (1.2-3.0 mL/kg/h).

### 5.2 Treatment at home and school

At home and in school, severe hypoglycemia should be immediately treated by administering glucagon. Glucagon can rapidly reverse severe hypoglycemia, except in situations of liver glycogen depletion after prolonged fasting, where intravenous administration of glucose solutions is more effective (48).

Children and adolescents with T1D of all ages with severe hypoglycemia can be treated with intramuscular (IM) or subcutaneous (SC) injection of recombinant crystalline glucagon available as a lyophilized (freeze-dried) powder. This formulation needs to be reconstituted to a concentration of 1 mg/mL with sterile water in a series of multiple steps immediately prior to injection. Two commercial glucagon rescue kits are currently available in Italy: GlucaGen^®^ HypoKit 1 mg (Novo Nordisk^®^A/S, Bagsvaerd, Denmark) and Glucagon Emergency Rescue Kit (Baqsimi, formerly Eli Lilly and Company, Indianapolis IN, USA, now Amphastar Pharmaceuticals, Inc., Rancho Cucamonga, CA, USA). The recommended dose of glucagon depends on body weight: adults and children >25 kg should receive 1 mg, whereas children should be treated with 0.5 mg. Nasal glucagon can be used from 4 years of age and above.

In recent years, intranasal (IN) glucagon has been increasingly used due to its easier administration. The IN formulation (Baqsimi™) is composed of glucagon as a powder with beta-cyclodextrin plus dodecylphosphocholine as the promoter for nasal absorption. Clinical trials have demonstrated that administration of IN glucagon is safe and effective for raising blood glucose levels during moderate hypoglycemia episodes under controlled conditions in adults (49) and children (50) with T1D, without being affected by the common cold and concomitant administration of nasal decongestant. Moreover, simulation studies have shown that the administration of nasal glucagon is faster and easier than injectable glucagon (51). The recommended dose of IN glucagon is 3 mg, equal to one puff. A recent meta-analysis demonstrated that intranasal glucagon and subcutaneous (SC)/intramuscular glucagon were equally effective for treating hypoglycemia (52). Moreover, additional real-life evidence has demonstrated the efficacy of IN glucagon in a large cohort of Italian children and adolescents (53). Common side-effects of IM and SC recombinant crystalline glucagon are nausea and vomiting and, in addition to these known side-effects, nasal glucagon may cause headache, upper airway discomfort, or nasal congestion (53).

### 5.3 Treatment of mild-to-moderate hypoglycemia

**Figure 1:**
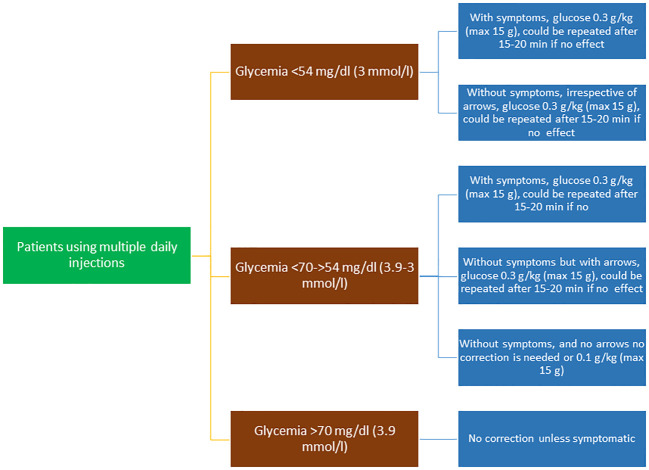
Flow chart of the treatment of hypoglycemic episodes in patients using multiple daily injections.

Mild-to-moderate hypoglycemia should be treated with rapidly absorbed carbohydrates. Subjects should re-test and re-ingest carbohydrates every 15 minutes until they recover from hypoglycemia (**Figure 1**).

The recommended dose of carbohydrate for children and adolescents is 0.3 g/kg oral glucose, which has been shown to be effective in increasing glucose levels by around 36 mg/dL within 15 minutes of ingestion in children (54). This approach was also found to be effective in children on insulin pumps (55).

Glucose-containing products (e.g., Glucosprint, Fastup, etc) are more effectively and quickly increase glucose levels than sucrose and fructose-containing products (56, 57). Glucose-containing tablets or drinks are currently not reimbursed by the National Health System in Italy, and they are more expensive than non-glucose-containing products. For these reasons, dietary sucrose and fructose-containing products (e.g., candy, sugar cubes, juice) are more frequently recommended in daily clinical practice. Both sucrose and fructose are required in greater amounts to provide the same increase in blood glucose compared with oral glucose (e.g., 20 g in the form of glucose tablets corresponds to 40 g of juice). Moreover, the rise in glycemia after sucrose ingestion is around half as fast as after glucose ingestion and could be even slower with fructose (56).

Parents of toddlers sometimes use honey and milk, but this approach should be discouraged: honey has a fructose content of around 70%, and the total sugar content is highly variable. Milk contains approximately 5 g of carbohydrate in 100 mL, and it causes a minimal rise in glycemia (around 4-5 mg/dL) (58). Complex carbohydrates and foods containing fats (e.g., chocolate) should also be avoided due to delayed intestinal absorption and the slow rise in glucose obtained.

### 5.4 Mini-dose glucagon

In cases of nausea, vomiting, or food refusal, small doses of subcutaneous glucagon can be administered to restore plasma glucose to normal. Using a standard U-100 insulin syringe, two units (20 μg) for children ≤ 2 years and 1 unit/year for children aged 3-15 years to a maximum dose of 150 μg or 15 units can be administered subcutaneously. If the blood glucose does not increase within 30 minutes, the initial dosage can be repeated. These dosage and treatment protocols are safe and effective in children, including toddlers, and increase glucose by 60-90 mg/dL within 30 minutes of administration (59).

### 5.5 Hypoglycemia treatment at school

School personnel should receive an appropriate diabetes education program to identify hypoglycemia signs and symptoms. In the “Hypoglycemia” section of the Diabetes Management Plan, it is essential to define individual signs and glycemic values that define intervention and glucagon use (60). Blood glucose meters should be available in school as a “first aid hypoglycemia management pack” containing glucose, glucose tablets, fast-acting sugar sources, and extra snacks, which should be available in the classroom or the bag of the child or adolescent with T1D.

### 5.6 Treatment using AID systems

Although AID systems have been shown to reduce episodes of hypoglycemia (19, 20, 28), they do not completely avoid them. Automated systems can reduce insulin delivery to zero for a period such that, during a hypoglycemia episode, there is usually less active insulin than with traditional insulin pump therapy. However, using these advanced systems, an excess of insulin and consequent hypoglycemia due to human mistakes (for example, a wrong bolus dose) or an unplanned intense physical activity can occur.

Although the traditional oral treatment of mild-to-moderate hypoglycemia in a conscious child involves a correction of 0.3 g/kg of glucose in a PWD using an insulin pump integrated with advanced algorithms, this correction is not always required. It is only necessary in a hypoglycemia alert in symptomatic subjects or within two to three hours of a bolus with excess active insulin. In real life, the current approach is correction with simple sugar/glucose at a dosage of 0.1 g/kg, resulting in 4-8 g of total carbohydrates, but waiting at least 15 minutes before treating to avoid glucose value oscillations. This practical advice arises from two considerations: these systems, in particular AID systems, continue to intervene in the administration of insulin, and subjects are significantly less insulinized than with conventional therapy; therefore, given that the system is reactive to blood sugar, if a sudden increase is detected (hypercorrection), an automatic corrective bolus is delivered that can help propagate new hypoglycemia (61, 62). Moreover, AID systems and sensors in general significantly reduce both severe and mild-to-moderate hypoglycemia episodes (61, 62), especially in the context of a structured educational program (62).

**Figure 2:**
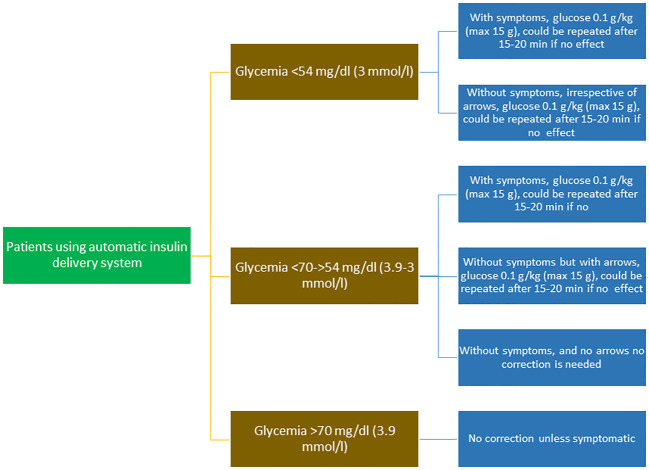
Flow chart of the treatment of hypoglycemic episodes in patients using automatic insulin delivery systems.

A summary of recommendations for hypoglycemia treatment in children and adolescents using AID systems is shown in **Table 4** and **Figure 2**.

**Table 4:** Recommendations for hypoglycemia treatment in patients using HCL/AHCL systems.

**WHEN**	>15 minutes at <70 mg/dL, hypoglycemia alert event
**START**	Treatment with 4-8 g carbs (0.1-0.15 g/kg)
**EXCEPTIONS** (more carbs)	- Hypoglycemia with exercise - When the PWD suspects a significant overestimation of carbs/meal bolus
**WAIT**	15 minutes before re-treating hypoglycemia to avoid oscillating glucose levels

Since alterations in glucose homeostasis might reduce gray and white matter volume and alter brain metabolism, it is important to note that hyperglycemia more than hypoglycemia can damage the brain. This is particularly interesting in relation to the therapeutic habit of preferring higher blood sugar levels in children with T1D rather than risk unwanted hypoglycemia. In this respect, using an AID system, especially in young children with T1D, can help to prevent both hypo- and hyperglycemia (63).

## 6 Prevention

Hypoglycemia is preventable because it is frequently predictable (1). Approaches to preventing hypoglycemia include glucose monitoring, patient education, meal planning, insulin therapy adjustment, glucose sensors, and AHCL pumps. To prevent hypoglycemia, diabetes education is essential. All children and adolescents and their caregivers should be educated on the risk factors for hypoglycemia to alert them to the times and situations where increased glucose monitoring is required and when treatment regimens need to be adjusted (3). For documented hypoglycemia without symptoms or impaired awareness of hypoglycemia, parents should contact their diabetes care team to review the care plan. To prevent hypoglycemia, it is extremely important to set AHCL systems to “activity” or “exercise” mode or set a temporary basal rate. These settings must be reevaluated periodically.

To prevent hypoglycemia, it is essential to encourage regular meal consumption and teach PWD how to count the carbohydrates contained in foods to administer the correct insulin dose. In addition to carbohydrates, fat and alcohol intake can influence glycemic trends. An excessive amount of fat in a meal slows down digestion and gastric emptying, thus making the insulin-carbohydrate association difficult to predict. Excessive fat favors the onset of hypoglycemia within the two hours following a meal and can delay hyperglycemia. Therefore, adequate food education is necessary to promote regular meal consumption and to learn how to count carbohydrates and calculate insulin doses. A suggestion for PWD and families is, therefore, to 1) follow their meal plan, 2) eat at least three evenly spaced meals each day with between-meal snacks as prescribed, and 3) plan meals no more than four to five hours apart. Families and caregivers of young PWD also need education on the risk factors for hypoglycemia so that they know when increased glucose monitoring is required and when treatment regimens need to be changed. Glucose monitoring using either flash or CGM should be performed before exercise, and extra carbohydrates may be consumed based on the glucose level and the expected exercise intensity and duration. Blood glucose targets may need to be adjusted upwards in children, adolescents, or young adults with diabetes with recurrent hypoglycemia and/or impaired hypoglycemia awareness.

## 7 Morbidity and mortality

### 7.1 Morbidity from hypoglycemia

Interest in the role of hypoglycemia as a cause of long-term morbidity has diminished. Exposure to chronic and repetitive hyperglycemia is now seen as a significant cause of permanent brain damage (64, 65). Transient cognitive dysfunction secondary to an episode of hypoglycemia is followed by a generally complete recovery within one hour of correction of low glucose levels. However, recovery from severe events can take up to 36 hours (66).

### 7.2 Mortality from hypoglycemia

Hypoglycemia has been proposed as a possible cause of “*death in bed*”, which seems to be more frequent in children with T1D than in the healthy population (67). However, it is difficult to demonstrate a causal link between the hypoglycemic event and the cause of death; even the recent ISPAD hypoglycemia guidelines (1) attribute the fatal event mainly to arrhythmic causes, autonomic neuropathy, and genetic predisposition (68–70), not hypoglycemia *per se*. For these reasons, it is wiser to certify death as a concurrent series of causes that include hypoglycemia.

## 8 Fear of hypoglycemia and psychological impact

Fear of hypoglycemia (FoH) is a fear that affects the quality of life and diabetes outcomes in PWD (68). While adequate concern about hypoglycemia is functional for good glucose management, FoH is a specific and extreme fear evoked by the risk and/or occurrence of low blood glucose levels.

Despite hypoglycemia still being a significant impediment to glycemic control, especially in the pediatric age range, the inappropriate self-management of some PWD may be due to FoH rather than the hypoglycemia itself (71). Fear of hypoglycemia can lead to an excessive delay in the administration of boluses with negative effects on metabolic control (72). Furthermore, FoH reduces the propensity for physical activity (73).

FoH is a phenomenon that involves both parents and children. Frequently, subjects and caregivers attribute their high level of anxiety about hypoglycemia to previous severe hypoglycemic episodes (74). The impact of FoH on PWD manifests both at the psychological level and in glycemic control due to behavioral avoidance and affective distress. Symptoms of hypoglycemia can compromise social lives, and FoH can be so strong that PWD avoid social activities. Moreover, the potential risk of hypoglycemia can lead to excessive vigilance in glucose management, with high anxiety levels (75). Progressive and persistent increases in HbA1c after episodes of severe hypoglycemia have been described (76, 77). Routine screening for FoH is vital to identify those who would benefit from intervention (77).

### 8.1 Predictors of fear of hypoglycemia

While the strongest predictor of parental FoH is the experience of a severe hypoglycemic event with their child, fear can occur without previous hypoglycemia (77). FoH may be neither consciously perceived nor explicitly declared. Therefore, clinicians need to suspect and seek it out and have an especially high index of suspicion for “over-compensatory behaviors” (e.g., decreasing in insulin dosage or snacking), “avoidance behaviors” (e.g., limiting physical or social activities), acceptance of persistently high blood glucose levels, excessive daily blood glucose checks, and not implementing “agreed” treatment changes to lower blood glucose levels.

### 8.2 Measurement of FoH

A commonly used screening tool for FoH is the Hypoglycemia Fear Survey (HFS), which has been adapted for parents as the Hypoglycemia Fear Survey-Parents (78) and parents of young children as well as adolescents and children themselves (79, 80). The Children’s Hypoglycemia Index (CHI) is another scale that has the added benefit of assessing FoH in specific situations such as only at night or school (80).

### 8.3 Fear of hypoglycemia and technologies

Advanced AID systems help to reduce nocturnal hypoglycemia, time spent in hypoglycemia, hyperglycemic episodes, and patient discomfort without increasing the risk of diabetic ketoacidosis or severe hypoglycemia (81). However, studies have not always reported greater satisfaction with treatment and a reduction in FoH with these systems (81). Patients who discontinued CGM showed a worsening of HbA1c levels compared with those who continued with CGM, who had a reduced FoH without improvements in glycemic levels (82). CGM systems with predictive alarms might reduce the time spent in hypoglycemia after physical activity (83), thereby contributing to the propensity for physical activity in PWD. Some believe that improvements in sleep quality and QoL in children and parents using these technologies are attributable to easier night-time control (84). Finally, the use of advanced technologies result in significant improvements in parents’ and children’s sleep quality and in parents’ FoH (85). The advancement and widespread deployment of such technologies has the potential to improve mental and physical health among PWD (86).

It is important to evaluate the psychosocial needs of young people with diabetes and their families when patients start using AID systems and during follow-up (87), establishing realistic expectations about the pros and cons of AID systems (86). Therefore, technological advances must be accompanied by well-timed training and adequate and continuous support (87, 88) and education.

### 8.4 Therapeutic interventions for FoH

Cognitive behavioral therapy and psychoeducational approaches have been shown to reduce this fear in adults. However, no studies have focused on children and adolescents, although these interventions may benefit older children (89). FoH contributes to the increased frequency of anxiety and depression in PWD and must be evaluated with a structured approach that includes specific screenings. Training must be individualized and take into account that those who have limited access to food due to cost also have limited options for dealing with hypoglycemia (90). A careful evaluation must also consider the coexistence of FoH in disadvantaged families (90).

The opinions of healthcare workers established using Delphi methodology indicated a need for specialized and expert staff (specialized nurses, educators) to train PWD on technologies applied to diabetes (91). Behavioral interventions for family members have shown persistent psychosocial benefits. CGM-focused education with behavioral support probably helps parents of young children with T1D reduce short- and long-term burden and worries (91). Furthermore, a telehealth approach may be helpful in the treatment of FoH (92).

## 9 Impaired awareness of hypoglycemia

Impaired awareness of hypoglycemia (IAH) is defined as the failure to perceive the appearance of autonomic warning symptoms and, consequently, the loss of ability to detect the onset of hypoglycemia and treat it promptly (93). IAH is associated with an approximately six-fold increased risk of developing severe hypoglycemia and represents a significant barrier to achieving optimal therapeutic goals. Four validated methods for assessing IAH are currently recognized: Clarke score, Gold score, Pedersen method, and HypoA-Q (94–97). Of these, the more detailed Clarke method seems to have higher specificity and accuracy for predicting the risk of clinically significant hypoglycemia (73) and, therefore, is preferred. CGM systems are also valuable tools for diagnosing IAH, especially if combined with one of the validated methods (98, 99).

According to epidemiological studies, the prevalence of IAH in children and adolescents with T1D assessed by the Clarke questionnaire varies from 16% to 22.4% (94, 100, 101). Younger age is the most reported factor associated with impaired hypoglycemia awareness (102–104).

IAH is also hypothesized to have a neurological component to its pathogenesis. Some brain regions, including the left amygdala and bilateral ventral striatum, show attenuated activation during hypoglycemic episodes, suggesting habituation of higher behavioral responses to hypoglycemia as a basis for unawareness (105). Recurrent hypoglycemia may be related to increased γ-aminobutyric acid inhibitory tone in the ventromedial hypothalamus and, thus, may be considered a mediator of hypoglycemia-associated autonomic failure (106, 107).

### 9.1 Restoring hypoglycemia unawareness

As the most critical risk factor for IAH is recurrent antecedent hypoglycemia, it is reasonable that the most crucial goal should be to reduce the incidence of hypoglycemia. Good metabolic control does not appear to increase the risk of unrecognized hypoglycemia, which is often associated with severe hypoglycemia and FoH (108).

Avoiding hypoglycemia interrupts the vicious cycle that impairs the ability of the adrenal medulla to produce epinephrine (a minor component of the counterregulatory response to hypoglycemia, which is primarily due to sympathetic neural activation) in response to blood glucose levels, restoring hypoglycemia awareness (109).

Technology can also help to restore hypoglycemia awareness; indeed, CGM is associated with a significant reduction in time spent in hypoglycemia episodes. Moreover, stopping insulin infusions when a low blood glucose value is encountered helps to avoid hypoglycemia and to re-start hypoglycemic symptoms. However, an unexpected limitation to restoring hypoglycemia awareness is that adolescents show a high acoustic arousal threshold from sleep (98), so they commonly continue to sleep through an alarm. Structured education on insulin administration, hypoglycemia training, blood glucose targets, and exercise management have also been shown to improve awareness of hypoglycemia (110–112).

## 10 Executive summary and recommendations

This document provides a series of clinical recommendations to prevent and treat hypoglycemia in children and adolescents with diabetes. All authors have long experience in the specialty and are members of the ISPED.

**Overview**

- Insulin-induced hypoglycemia and fear of hypoglycemia (FoH) are major limiting factors in glycemic management and a significant concern for children and adolescents with diabetes and their caregivers.
- Hypoglycemia is defined by autonomic or neuroglycopenic symptoms, low plasma glucose levels (<70 mg/dL), and symptomatic response to carbohydrate administration.
- Symptoms of hypoglycemia result from adrenergic activation (palpitations, sweating, shaking sensation) and neuroglycopenia (headache, drowsiness, difficulty concentrating). Younger children may exhibit behavioral changes such as irritability, restlessness, calmness, and tantrums.
- Three clinical levels of hypoglycemia are recognized:
  ○ Level 1 – Clinical hypoglycemia alert
  A glucose value of <3.9 mmol/L (70 mg/dL) is an alert value that requires attention to prevent more severe hypoglycemia. The alert can be used as the threshold value for identifying and treating hypoglycemia in children with diabetes due to the potential for glucose levels to drop further.
  ○ Level 2 - Clinically important or severe hypoglycemia
  Glucose values <3.0 mmol/L (54 mg/dL) indicate clinically significant or serious hypoglycemia. These low levels may lead to defective hormonal counter-regulation and impaired awareness of hypoglycemia (IAH). Neurogenic symptoms and cognitive dysfunction occur below this level, with a subsequent increased risk of severe hypoglycemia.
  ○ Level 3 – Severe hypoglycemia
  Severe hypoglycemia is an event associated with severe cognitive impairment (including loss of consciousness and seizures) that requires the assistance of another person to administer intravenous carbohydrates, glucagon, or glucose.
- Children with diabetes can experience impaired hypoglycemia awareness and, when present, it is associated with a significantly increased risk of severe hypoglycemia.
- There have been significant reductions in the incidence rates of severe hypoglycemia over the past two decades for several reasons, not least the introduction of insulin analogues, improved diabetes technologies, and improved hypoglycemia education.
- Younger children often exhibit non-specific and behavioral symptoms due to combined adrenergic and neuroglycopenic responses, so the observed signs are more important than symptoms.
- Transient cognitive dysfunction secondary to a hypoglycemic episode is usually followed by complete recovery within one hour of correction of low glucose levels. However, recovery from severe events can take up to 36 hours. There is currently no high-quality evidence on the impact of hypoglycemia on lifelong cognitive impairment.
- Currently, available technologies such as continuous glucose monitoring (CGM), predictive low glucose management (PLGM), and automated insulin delivery (AID) systems reduce the frequency and duration of hypoglycemic episodes.
- Modifiable hypoglycemic risk factors include the type of insulin treatment and doses, physical activity, diet and alcohol habits, and drug use or substance abuse.
- The risk of hypoglycemia is greater with aerobic than anaerobic exercise. Nighttime hypoglycemia following exercise is mainly due to depletion of glucose stores, impaired counter-regulatory hormone responses during sleep, and increased insulin sensitivity due to nighttime fasting.
- Celiac disease, Addison’s disease, and hypothyroidism should be considered in children with unexplained hypoglycemia.

**Management**

- The goal of treatment is to raise blood glucose above 70 mg/dL and to prevent further decreases.
- Oral glucose at a dose of 0.3 g/kg is the preferred treatment for the conscious individual with blood glucose <70 mg/dL (3.9 mmol/L), although any form of carbohydrate containing glucose may be used. Blood sugar levels increase about twice as fast with glucose than with sucrose (**Figure 1**).
- In patients treated with AID systems, non-severe hypoglycemia does not always need to be corrected. Correction is only needed for a hypoglycemia alert in symptomatic patients or within two to three hours of a bolus with an excess of active insulin, and usually a glucose dose of 0.1 g/kg is enough to raise glycemia above a safe level (**Figure 2**).
- Severe hypoglycemia occurring at home or school should be treated immediately with subcutaneous or intranasal glucagon.
- To treat severe hypoglycemia in a hospital setting, intravenous glucose (recommended dose 0.2 g/kg) must be administered immediately to limit exposure to hypoglycemia. Highly concentrated glucose solutions (50%) or infusion rates >5 mg/kg/min should be avoided due to the risk of excessive rate of osmotic change and, consequently, hyperosmolar cerebral injury.

**Prevention**

- Hypoglycemia should be prevented, as it is associated with severe physical and psychological distress in both patients and caregivers.
- Education and diabetes technologies are the primary tools for preventing hypoglycemia.
- Extending diabetes education to parents, schoolteachers, and other health professionals is a priority so that they can recognize early warning signs of hypoglycemia and treat low blood glucose immediately and appropriately.
- Glucose monitoring should be performed before physical activity, and carbohydrate correction should be performed as needed. Oral glucose should always be available during exercise.
- Adjusting insulin doses and changing glycemic targets may be necessary in children with frequent hypoglycemia.
- For documented hypoglycemia without symptoms or impaired awareness of hypoglycemia, parents should contact their diabetes care team to review the care plan.
- Impaired awareness of hypoglycemia should be routinely tested in clinical practice. Clarke, Gold, or Pedersen-Bjergaard scores are useful for assessing impaired awareness. Reductions in hypoglycemia episodes may reduce impaired awareness of hypoglycemia.
- All children and adolescents with diabetes should be prescribed intranasal or subcutaneous glucagon. Patients, parents, and caregivers must be trained in its use.
- Periodic screening of children and parents for FoH helps to identify cases where more educational intervention is needed.

**Future directions**

- It is important to identify gaps in the skills and self-efficacy of children and adolescents with T1D and their families, especially when from a different cultural, socioeconomic, or educational background together with other perceived enablers of, and barriers to, self-management in this population.
- Specific educational paths are essential to help these people to correctly manage, treat, and prevent hypoglycemia episodes.
- Diabetes healthcare stakeholders may consider strategies for regular educational reinforcement in patients to foster healthy coping with diabetes stress, exercise planning to avoid hypoglycemia, interpreting blood glucose patterns, and adjusting medications or foods to reach target blood glucose levels.
- Furthermore, designing interventions that capitalize on how to use relevant technological devices could enhance diabetes self-management.

## Author contributions

SZ: Conceptualization, Methodology, Supervision, Validation, Writing – original draft, Writing – review & editing. ST: Methodology, Supervision, Validation, Writing – review & editing, Data curation, Investigation. AS: Conceptualization, Data curation, Investigation, Supervision, Validation, Visualization, Writing – original draft, Writing – review & editing. RB: Conceptualization, Supervision, Validation, Writing – review & editing. MD: Data curation, Investigation, Writing – review & editing. RF: Data curation, Investigation, Writing – review & editing. DI: Data curation, Investigation, Writing – review & editing. LL: Data curation, Investigation, Writing – review & editing. EM: Data curation, Investigation, Writing – review & editing. SP: Data curation, Investigation, Writing – review & editing. CP: Data curation, Investigation, Writing – review & editing. IR: Data curation, Investigation, Writing – review & editing. NR: Data curation, Investigation, Writing – review & editing. AR: Data curation, Investigation, Writing – review & editing. CR: Data curation, Investigation, Writing – review & editing. GS: Data curation, Investigation, Writing – review & editing. SS: Data curation, Investigation, Writing – review & editing. RS: Data curation, Investigation, Writing – review & editing. AZ: Data curation, Investigation, Writing – review & editing. VC: Conceptualization, Data curation, Investigation, Methodology, Supervision, Validation, Writing – original draft, Writing – review & editing.

## Group members of Diabetes Study Group of the Italian Society for Pediatric Endocrinology and Diabetes

Albino Claudia Accursia, Aloe Monica, Anzelotti Maria Teresa, Arnaldi Claudia, Barbetti Fabrizio, Bassi Marta, Berioli Maria Giulia, Bernardini Luca, Bertelli Enrica, Biagioni Martina, Bobbio Adriana, Bombaci Bruno, Bonfanti Riccardo, Bonura Clara, Bracciolini Giulia Patrizia, Bruzzese Mariella, Bruzzi Patrizia, Buono Pietro, Buscarino Piera, Cadario Francesco, Calcaterra Valeria, Calzi Elena, Cappa Marco, Cardani Roberta, Cardella Francesca, Cardinale Giuliana Marcella, Casertano Alberto, Castorani Valeria, Cauvin Vittoria, Cenciarelli Valentina, Ceruti Franco, Cherubini Valentino, Chiarelli Francesco, Chiari Giovanni, Cianfarani Stefano, Cicchetti Mario, Cipriano Paola, Cirillo Dante, Citriniti Felice, Coccioli Maria Susanna, Confetto Santino, Contreas Giovanna, Coro Anna, Correddu Antonella, Corsini Elisa, Crino’ Antonino, d’Annunzio Giuseppe, De Berardinis Fiorella, De Donno Valeria, De Filippo Gianpaolo, De Marco Rosaria, De Sanctis Luisa, Del Duca Elisabetta, Delvecchio Maurizio, Deodati Annalisa, Di Bonito Procolo, Di Candia Francesca, Faleschini Elena, Fattorusso Valentina, Favia, Anna, Federico Giovanni, Felappi Barbara, Ferrari Mara, Ferrito Lucia, Fichera Graziella, Fontana Franco, Fornari Elena, Franceschi Roberto, Franco Francesca, Franzese Adriana, Frongia Anna Paola, Frontino Giulio, Gaiero Alberto, Galassi Sabrina Maria, Gallo Francesco, Gargantini Luigi, Giani Elisa, Gortan Anna Jolanda, Graziani Vanna, Grosso Caterina, Gualtieri Antonella, Guasti Monica, Guerraggio Lucia Paola, Guzzetti Chiara, Iafusco Dario, Iannicelli Gennaro, Iezzi Maria Laura, Ignaccolo Maria Giovanna, Innaurato Stefania, Inzaghi Elena, Iovane Brunella, Iughetti Lorenzo, Kaufmann Peter, La Loggia Alfonso, Lambertini Anna Giulia, Lapolla Rosa, Lasagni Anna, Lazzaro Nicola, Lazzeroni Pietro, Lenzi Lorenzo, Lera Riccardo, Levantini Gabriella, Lezzi Marilea, Lia Rosanna, Liguori Alice, Lo Presti Donatella, Lombardo Fortunato, Lonero Antonella, Longhi Silvia, Lorubbio Antonella, Lucchesi Sonia, Maccioni Rosella, Macedoni Maddalena, Macellaro Patrizia Cristiana, Madeo Simona Filomena, Maffeis Claudio, Mainetti Benedetta, Maltoni Giulio, Mameli Chiara, Mammì Francesco, Manca Bitti Maria Luisa, Mancioppi Valentina, Manco Melania, Marigliano Marco, Marino Monica, Marsciani Alberto, Matteoli Maria Cristina, Mazzali Elena, Minute Marta, Minuto Nicola, Monti Sara, Morandi Anita, Morganti Gianfranco, Morotti Elisa, Mozzillo Enza, Musolino Gianluca, Olivieri Francesca, Ortolani Federica, Pampanini Valentina, Pardi Daniela, Pascarella Filomena, Pasquino Bruno, Passanisi Stefano, Patera Ippolita Patrizia, Pedini Annalisa, Pennati Maria Cristina, Peruzzi Sonia, Peverelli Paola, Pezzino Giulia, Piccini Barbara, Piccinno Elvira Eugenia Rosaria, Piona Claudia, Piredda Gavina, Piscopo Alessia, Pistone Carmelo, Pozzi Erica, Prandi Elena, Predieri Barbara, Prudente Sabrina, Pulcina Anna, Rabbone Ivana, Randazzo Emioli, Rapini Novella, Reinstadler Petra, Riboni Sara, Ricciardi Maria Rossella, Rigamonti Andrea, Ripoli Carlo, Rossi Virginia, Rossi Paolo, Rutigliano Irene, Sabbion Alberto, Salvatoni Alessandro, Salvo Caterina, Salzano Giuseppina, Sanseviero Mariateresa, Savastio Silvia, Savini Rosanna, Scanu Mariapiera, Scaramuzza Andrea Enzo, Schiaffini Riccardo, Schiavone Maurizio, Schieven Eleonardo, Scipione Mirella, Secco Andrea, Silvestri Francesca, Siri Giulia, Sogno Valin Paola, Sordelli Silvia, Spiri Daniele, Stagi Stefano, Stamati Filomena Andreina, Suprani Tosca, Talarico Valentina, Tiberi Valentina, Timpanaro Tiziana Antonia Lucia, Tinti Davide, Tirendi Antonina, Tomaselli Letizia Grazia, Toni Sonia, Torelli Cataldo, Tornese Gianluca, Trada Michela, Trettene Adolfo Andrea, Tumini Stefano, Tumminelli Marilena, Valerio Giuliana, Vandelli Sara, Ventrici Claudia, Zampolli Maria, Zanatta Manuela, Zanfardino Angela, Zecchino Clara, Zonca Silvia, Zucchini Stefano.
